# Neural circuits for the adaptive regulation of fear and extinction memory

**DOI:** 10.3389/fnbeh.2024.1352797

**Published:** 2024-02-02

**Authors:** Samantha L. Plas, Tuğçe Tuna, Hugo Bayer, Vitor A. L. Juliano, Samantha O. Sweck, Angel D. Arellano Perez, James E. Hassell, Stephen Maren

**Affiliations:** ^1^Department of Psychological and Brain Sciences, Texas A&M University, College Station, TX, United States; ^2^Institute for Neuroscience, Texas A&M University, College Station, TX, United States; ^3^Department of Pharmacology, Institute of Biomedical Sciences, University of São Paulo, São Paulo, Brazil

**Keywords:** fear memory, extinction, prefrontal cortex, hippocampus, amygdala, rat

## Abstract

The regulation of fear memories is critical for adaptive behaviors and dysregulation of these processes is implicated in trauma- and stress-related disorders. Treatments for these disorders include pharmacological interventions as well as exposure-based therapies, which rely upon extinction learning. Considerable attention has been directed toward elucidating the neural mechanisms underlying fear and extinction learning. In this review, we will discuss historic discoveries and emerging evidence on the neural mechanisms of the adaptive regulation of fear and extinction memories. We will focus on neural circuits regulating the acquisition and extinction of Pavlovian fear conditioning in rodent models, particularly the role of the medial prefrontal cortex and hippocampus in the contextual control of extinguished fear memories. We will also consider new work revealing an important role for the thalamic nucleus reuniens in the modulation of prefrontal-hippocampal interactions in extinction learning and memory. Finally, we will explore the effects of stress on this circuit and the clinical implications of these findings.

## 1 Introduction

Understanding the neural mechanisms mediating fear and extinction learning is vital for the development of novel therapeutics for fear and anxiety-related disorders such as phobias, post-traumatic stress disorder (PTSD), and obsessive-compulsive disorder. Exposure-based therapies are commonly used for treating these disorders and extinction learning is the underlying mechanism. Critically, patients undergoing such therapies are vulnerable to relapse because extinction memories fade with time and are context-dependent (Maren et al., 2013; Bouton et al., 2021). Given the prevalence of these disorders and the limitations of exposure-based therapies, it is essential to understand the neural mechanisms of these disorders.

An important behavioral model for this work is Pavlovian fear conditioning, in which a conditioned stimulus (CS) is paired with an aversive unconditioned stimulus (US) (e.g., footshock), ultimately leading to a conditioned response (CR), such as freezing behavior. After conditioning, CRs can be extinguished by repeatedly exposing rats to the CS in the absence of the US. This procedure reliably suppresses the magnitude and probability of fear CRs, but this outcome is fragile. Extinction is highly vulnerable to several forms of relapse such as renewal, reinstatement, and spontaneous recovery (Bouton et al., 2021). In this review, we will review decades of work revealing a critical role of the amygdala, hippocampus (HPC) and medial prefrontal cortex (mPFC) in the acquisition, expression, and retrieval of fear. Additionally, we will explore the role of the HPC and its interactions with the infralimbic cortex in the acquisition and expression of extinction memories. Importantly, we will address the critical point that extinction learning does not erase original fear memories but creates a competing extinction memory. From this perspective, the inhibition of fear after extinction requires the suppression of the fear memory in addition to the retrieval of the extinction memory. This process is mediated by prefrontal-hippocampal interactions that are coordinated by the thalamic nucleus reuniens (RE). Finally, we will discuss the vulnerability of these processes to stress and how this contributes to PTSD-like phenotypes.

## 2 Neural circuits for the acquisition of fear memory

Decades of work assessing the neural circuitry of fear learning point to the amygdala as a critical neural substrate of aversive learning and memory. The amygdala is an almond shaped structure located in the temporal lobe that is essential for the acquisition and expression of conditioned fear. During fear conditioning, sensory inputs converge in the basolateral amygdala (BLA) (LeDoux et al., 1990; Romanski et al., 1993), driving plastic changes and allowing for long-term associations to be formed (Rogan et al., 1997; Quirk et al., 1995). Primary sensory information about unimodal CSs reaches the BLA through both thalamic and cortical projections, whereas multimodal information about the context is conveyed by the HPC and entorhinal cortex (Maren, 2001). Information about aversive (e.g., shock) USs appears to involve projections from the parabrachial nucleus (Bernard et al., 1993; Davis et al., 1994). The long-term synaptic changes in the BLA that underlie fear conditioning are mediated by *N*-methyl-D-aspartate receptors (NMDAR) (Maren et al., 1996; Fendt, 2001; Rodrigues et al., 2001; Goosens and Maren, 2004). Projections from the BLA to the central nucleus of the amygdala (CeA) drive the expression of conditioned fear responses (Goosens and Maren, 2001; Jimenez and Maren, 2009; Ciocchi et al., 2010; Duvarci et al., 2011) through projections to different hypothalamic and brainstem structures, including projections to the periaqueductal gray for the freezing CR (LeDoux, 2000; Paré et al., 2004). The CeA also plays a role in the acquisition of conditional fear (Goosens and Maren, 2001, 2003; Wilensky et al., 2006; Ciocchi et al., 2010; Duvarci et al., 2011; Li et al., 2013).

The HPC plays a critical role in encoding contextual stimuli during fear conditioning. Hippocampal contextual representations are communicated to the BLA which then projects to the CeA for CRs (LeDoux, 2000; Maren, 2001; Kim and Cho, 2020). Early studies examining the dorsal HPC (dHPC) found that electrolytic lesions prevent the acquisition and expression of contextual fear, but not fear conditioning to auditory CSs (Kim and Fanselow, 1992; Phillips and LeDoux, 1992). In line with these findings, NMDAR antagonism in the dHPC yields similar results, suggesting that hippocampal NMDARs are involved in context conditioning (Young et al., 1994). Subsequent work revealed that dHPC lesions are most effective when made after fear conditioning (Maren et al., 1997), indicating that animals can acquire contextual fear conditioning using multiple strategies. In the absence of the dHPC, an elemental (rather than configural) representation of context may be sufficient to support contextual learning (Maren et al., 1997). The role of the HPC in contextual learning is confirmed by more recent studies using modern techniques such as optogenetics and engram tagging methods. For example, Kheirbek et al. (2013), use optogenetic approaches to demonstrate that the dorsal, but not ventral, dentate gyrus (DG) is required for the encoding of contextual fear memories. This work was expanded on by Bernier et al. (2017), who showed that optogenetically silencing the DG during contextual fear conditioning impaired the acquisition of contextual fear. In addition, optogenetic inhibition of the DG impaired fear expression in the conditioning context when the context was similar to a neutral context. On the other hand, inhibition of the DG enhanced fear generalization to the neutral context that was similar to the conditioning context. Collectively, these data point toward a role for the DG in the acquisition and recall of contextual fear (Bernier et al., 2017). Indeed, using engram tagging methods, it is demonstrated that contextual fear conditioning creates a memory trace in the dorsal DG and CA3 regions of the HPC (Denny et al., 2014; Cazzulino et al., 2016), and silencing these neurons blocks fear memory expression (Denny et al., 2014), while optogenetic activation of these engram cells in the dorsal DG is sufficient to promote fear memory recall (Liu et al., 2012; Kitamura et al., 2017). In addition to the role of the dorsal DG and CA3, it was found that optogenetically silencing dorsal CA1 during a contextual fear recall test impairs both recent and remote memory recall (Goshen et al., 2011). Silencing CA1 cells tagged during contextual fear conditioning-tagged also impairs memory retrieval (Tanaka et al., 2014). These studies further corroborate earlier studies demonstrating a role for the dHPC in fear memory acquisition and expression.

Contextual memories encoded by the HPC also involve the RE, a midline thalamic nucleus that interconnects the HPC and mPFC via bidirectional connections. Pharmacological inhibition of the RE impairs the acquisition of contextual fear conditioning and disrupts contextual discrimination (Xu and Südhof, 2013; Ramanathan et al., 2018b; Troyner et al., 2018). Interestingly, lost contextual memories can be restored by inactivating the RE during retrieval testing. This suggests that contextual fear memories learned when the RE is inactivated are state-dependent, and can only be retrieved when the RE is offline. Indeed, although contextual conditioning normally requires the dHPC, the dHPC is not required to form contextual memories with the RE offline (Ramanathan et al., 2018b).

**FIGURE 1:**
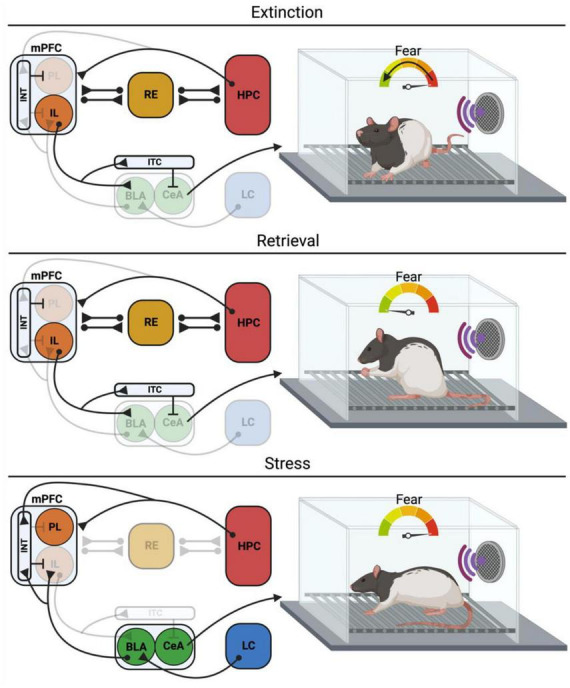
Neural circuits for extinction, retrieval and retrieval under stress. During extinction the IL exerts inhibitory control over the BLA and CeA, particularly through its inputs into the ITCs, thus suppressing the expression of the conditioned fear response. Concurrently, the HPC is encoding a configural representation of the extinction context, allowing for the contextual regulation of extinction learning and retrieval. Under retrieval conditions, the RE facilitates information flow from the mPFC to the HPC so that the mPFC can exert top-down control of the HPC, allowing for suppression of context inappropriate memories and retrieval of context appropriate memories. The IL also exerts inhibitory control of the BLA and CeA, suppressing the conditioned fear response. However, stress can impact these processes, resulting in impaired extinction retrieval. In the case of the immediate extinction deficit (IED), stress engages the LC-NE system, which excites the BLA through activation of β-adrenergic receptors. IL projecting BLA neurons drive feedforward inhibition of the IL through synapses on parvalbumin INT of the IL, impairing extinction retrieval. Other models of stress have demonstrated a role for the HPC in stress-induced extinction retrieval deficits, although the mechanisms and projections are not as clear. mPFC, Medial prefrontal cortex; PL, prelimbic; IL, infralimbic; INT, interneurons; BLA, basolateral amygdala; CeA, central amygdala; ITC, intercalated cells; RE, nucleus reuniens; HPC, hippocampus; LC, locus coeruleus. Created with BioRender.com.

The mPFC is also implicated in the acquisition and expression of Pavlovian fear conditioning (Giustino and Maren, 2015). The prevailing view is that the prelimbic (PL) and infralimbic (IL) regions of the mPFC have opposing roles in the regulation of fear (Quirk and Mueller, 2008). For instance, Corcoran and Quirk (2007) found that the PL is necessary for the expression, but not acquisition, of learned fear. Inactivation of the PL prior to fear extinction reduces fear expression but does not interfere with fear extinction. In contrast, inactivation of the IL does not affect fear expression but impairs extinction learning and retrieval (Sierra-Mercado et al., 2011; Marek et al., 2018). In the following sections, we will focus on the extinction of fear memories and the role of the mPFC, HPC, and mPFC-HPC interactions in the acquisition and retrieval of extinction memories (Figure 1).

## 3 Acquisition of extinction memory

Extinction of conditioned fear has generated considerable interest because it is a fundamental behavioral process underlying exposure therapy in humans. Extensive research has identified the amygdala as a key substrate in both acquisition and extinction of fear memories (LeDoux, 1995; Maren, 2001). In addition, the mPFC plays a critical role in extinction learning and the regulation of fear (Gilmartin et al., 2014; Giustino and Maren, 2015; Quirk and Mueller, 2008). Within the mPFC, the PL and IL are key players in these processes. Inputs to the IL and PL include excitatory projections from structures such as the HPC, BLA, midline thalamus, and contralateral mPFC (Krettek and Price, 1977). Most important for this current review are the projections from the HPC (Swanson, 1981; Jay et al., 1989). These projections are believed to transmit contextual information to the mPFC, which is critical for directing memory retrieval processes that guide context-appropriate behavioral responses (Preston and Eichenbaum, 2013). mPFC outputs include robust glutamatergic projections to the BLA, midline thalamus, and dorsomedial striatum (DeFelipe and Fariñas, 1992; Pinto and Sesack, 2000, 2008; Xu and Südhof, 2013). Despite several structural and connective similarities, a dichotomy has emerged in the functionality of the IL and PL in fear expression.

### 3.1 Infralimbic cortex is essential for extinction learning and memory

Considerable work has revealed that while the PL is involved in the production of conditioned fear responses, the IL serves to inhibit these responses after extinction training (Sotres-Bayon and Quirk, 2010). For example, lesions of the IL spare the acquisition of extinction (indexed by within-session reductions in fear CRs) but impair the subsequent retrieval of extinction memories (Quirk et al., 2000). Temporary pharmacological inactivation of the IL reduces both the acquisition and later retrieval of extinction memory (Sierra-Mercado et al., 2006, 2011; Laurent and Westbrook, 2009; Marek et al., 2018). Pharmacological manipulations of the IL immediately after extinction training, such as protein synthesis inhibition (Santini et al., 2004) or NMDAR antagonism (Sotres-Bayon et al., 2009), also impair extinction retrieval. Conversely, enhancing IL activity immediately after extinction training facilitates extinction retrieval (Santini et al., 2012). Chang and Maren (2011) also found that IL activation via application of a GABA_*A*_ antagonist or an NMDAR partial agonist immediately prior to extinction training reduces conditioned freezing and enhances extinction acquisition and retrieval. These studies suggest that IL participates in the acquisition, consolidation, and expression of extinction memories (Figure 1).

More recent work implicates IL principal cells and their projections in these processes. For example, optogenetic activation of IL pyramidal cells (Do-Monte et al., 2015) or their terminals in the amygdala (Bukalo et al., 2015) during extinction training facilitates later extinction retrieval. In contrast, optogenetically silencing IL pyramidal cells or IL-amygdala projection during extinction retrieval did not attenuate the extinction retrieval (Bukalo et al., 2015; Do-Monte et al., 2015). Though it is important to appreciate that the extinction retrieval tests in these studies were conducted with a limited number of trials. This may have promoted spontaneous recovery, a type of fear relapse that can emerge with mere passage of time, to occlude the effects of the manipulations. With extended retrieval tests, other studies have found that IL neurons are critical for extinction retrieval (Laurent and Westbrook, 2009; Kim et al., 2016; Marek et al., 2018).

### 3.2 Hippocampal involvement in extinction learning guides adaptive behavioral responses

Extinction memories are highly context dependent (Maren et al., 2013; Bouton et al., 2021) making them vulnerable to relapse and limiting the success of exposure-based therapies that are used to treat fear, anxiety, and trauma- and stressor-related disorders, such as PTSD. Once extinction of a trauma-related cue is attained, patients can lose the ability to regulate fear with the simple passage of time, through exposure to stressors, or exposure to the cue outside of the exposure-based therapy setting. These forms of recovery of fear or relapse can be modeled in Pavlovian fear conditioning and include spontaneous recovery in which fear re-emerges with mere passage of time (Pavlov, 1927; Rescorla, 2004), reacquisition refers to re-exposure to the CS-US pairing after extinction (Napier et al., 1992; Kehoe and Macrae, 1997; Rescorla, 2001; Bouton, 2002), and reinstatement which consists of exposure to US after extinction (Bouton, 2000, 2002, 2004; Maren et al., 2013; Goode and Maren, 2014; Chen et al., 2019). A fourth form of fear relapse known as renewal happens when the CS is encountered outside of the extinction context. This can occur in either the conditioning (ABA renewal) or a novel (ABC renewal) context (Boschen et al., 2009; for a review see Gunther et al., 1998; Vervliet et al., 2013; Goode and Maren, 2014; Bouton et al., 2021). Therefore, extinction memories are specific to the context in which they are acquired, and retrieval of these extinction memories requires context information. In other words, context serves as a cue to retrieve the appropriate memory associated with it (Bouton, 1993; Bouton and Ricker, 1994; Corcoran et al., 2005; Orsini and Maren, 2012). The fragility of the extinction memory when compared with the conditioning memory it competes with strongly suggests that extinction is not an erasure or modification of fear, but rather a separate memory that inhibits the conditioning memory during successful extinction retrieval (Bouton et al., 2006b; Vervliet et al., 2013).

Considerable work indicates that the context-dependence of extinction is mediated by the HPC (Orsini and Maren, 2012; Maren et al., 2013). The involvement of the HPC in the context-dependence of extinction is not surprising insofar as many studies in both humans and rodents have shown that the HPC is essential for contextual learning and memory (Kim and Fanselow, 1992; Maren et al., 1997; Anagnostaras et al., 1999; Lang et al., 2009; Poppenk et al., 2013). Numerous studies suggest that the HPC encodes configural representations of context in which several discrete sensory elements are assembled into a unified representation or a “gestalt-like” memory (Fanselow, 2000; Maren and Fanselow, 1995; Rudy and Sutherland, 1995). During extinction learning, there is evidence that NMDAR activation and *de novo* protein synthesis in the dHPC are required for the consolidation of extinction memories (Vianna et al., 2001, 2003). Similarly, dorsal hippocampal inactivation impairs acquisition of extinction (Corcoran et al., 2005). These findings suggest that the HPC is required for both the encoding and consolidation of extinction memories (Figure 1). Recent work with neuronal capture techniques has shown that distinct ensembles of dorsal hippocampal neurons or “engrams” can encode both fear and extinction memories (Lacagnina et al., 2019). These engrams are reactivated during memory retrieval and can either promote or attenuate conditional fear. We have recently shown, for example, that the retrieval of a contextual fear memory reactivates dorsal hippocampal ensembles active during fear conditioning. Fear memory ensembles captured during memory retrieval promote freezing behavior when chemogenetically reactivated (Ressler et al., 2021). Together, these studies suggest that hippocampal ensembles encode contextual representations that can both promote and attenuate conditional fear under different circumstances.

Once extinction is acquired, the expression of conditional fear is highly context-dependent (Maren et al., 2013; Bouton et al., 2021). For example, an extinguished CS elicits little freezing behavior in the extinction context, but the same CS will produce robust freezing in any other context. This relapse or “renewal” of extinguished fear depends on the HPC. Early studies showed that pharmacological inactivation of the dHPC prevents fear renewal (Corcoran and Maren, 2001; Hobin et al., 2006) and dampens the relapse-associated increases in the lateral amygdala activity that accompany renewal (Maren and Hobin, 2007). Renewal is associated with increased expression of the immediate early gene protein Fos in the ventral HPC (vHPC), particularly in neurons that project to the basal amygdala and PL (Orsini et al., 2011; Jin and Maren, 2015a; Wang et al., 2016). Disconnection of these projections (Orsini et al., 2011) attenuated renewal. Projections from the vHPC to the IL are particularly important for renewal (Marek et al., 2018). Chemogenetically activating these projections drives relapse of extinguished fear in the extinction context, whereas silencing these projections attenuated renewal outside the extinction context. These outcomes are paradoxical given the strong glutamatergic projection of the vHPC to IL—a projection one might imagine would suppress freezing by driving IL neurons that project to and inhibit the amygdala. However, it has been shown that ventral hippocampal neurons exert strong feed-forward inhibition on IL principal cells via parvalbumin (PV)-positive interneurons. Thus, renewal of extinguished fear results from hippocampal inhibition of mPFC circuits involved in the suppression of fear. When an extinguished CS is presented outside the extinction context, the expectation of safety is violated (Maren, 2014) and animals mount an adaptive fear response. The HPC is required for this process; encountering an extinguished CS in a novel context (or the conditioning context) restores the conditioning memory and drives hippocampal-prefrontal projections to attenuate extinction retrieval.

Ultimately, interactions between the HPC and mPFC are crucial for cognitive and emotional processes that underlie acquisition and expression of extinction memories (Figure 1). The mPFC is crucial for encoding and retrieval of extinction memories, relying on contextual information that is conveyed by the HPC to adjust behavior adaptively. Indeed, the mPFC and HPC are thought to be a part of a network of structures involved in encoding of information and contextual regulation of extinction learning and retrieval (Maren et al., 2013). The process by which the mPFC and HPC communicate during the retrieval of extinction memories is covered in the following section.

## 4 Retrieval of extinction memory and suppression of fear

### 4.1 Prefrontal-amygdala circuits mediating acquisition of fear extinction

Because extinction memories must compete with their associated conditioning memories, a degree of top-down control is necessary to determine when to suppress fear. Indeed, the mPFC, which exerts top-down control over multiple structures in the brain, is a major regulator of fear expression (Morgan et al., 1993; Quirk and Beer, 2006; Kim et al., 2010; Maren and Holmes, 2016). Traditional accounts of fear extinction address the projections from the mPFC to the BLA, with PL and IL showing differential control over fear expression. As mentioned above, PL drives fear expression (Corcoran and Quirk, 2007; Sierra-Mercado et al., 2011) and its activity is correlated with activity in the BLA, which is reactive to fearful stimuli (Tye et al., 2011) (Figure 1). The PL is also associated with context-dependent renewal of fear (Sharpe and Killcross, 2015; Vasquez et al., 2019). IL, conversely, drives fear inhibition during extinction acquisition by interacting with the inhibitory cells in the BLA (Milad and Quirk, 2002; Vertes, 2004; Sierra-Mercado et al., 2011), primarily through intercalated cell masses (ITC)s (Likhtik et al., 2008; Sotres-Bayon and Quirk, 2010) (Figure 1).

Studies exploring the role of IL in extinction retrieval have yielded inconsistent results (Arruda-Carvalho and Clem, 2015; Giustino and Maren, 2015; Kim et al., 2016), presumably due to the differences in techniques and their spatiotemporal precision, targets (e.g., IL-only vs. IL and PL), species, and behavioral paradigms (e.g., number of trials). Recently, several optogenetics studies sought to examine the role of the IL in extinction retrieval. In a series of studies, Do-Monte et al. (2015) used Pavlovian auditory fear conditioning paradigm in rats in combination with optogenetic manipulations to examine the role of the IL in extinction retrieval. They expressed the excitatory opsin channelrhodopsin (ChR2) in IL pyramidal neurons and stimulated this neuronal population during CSs in extinction training. Stimulation caused lower freezing during extinction and facilitated extinction retrieval in a light-free test next day. Conversely, silencing IL pyramidal neurons during extinction did not affect freezing during extinction but impaired extinction retrieval the following day. Silencing IL during extinction retrieval did not have an impact on freezing, suggesting that IL activity is not necessary for extinction retrieval (Do-Monte et al., 2015). Kim et al. (2016), on the other hand, showed that a similar photoinhibition of IL in mice during extinction retrieval CSs impairs extinction retrieval and increases freezing levels, indicating that IL is necessary to suppress fear expression after extinction (Kim et al., 2016). Pharmacological inhibition of IL also impairs extinction retrieval (Laurent and Westbrook, 2009; Marek et al., 2018).

Bukalo et al. (2015) examined the role of mPFC to amygdala pathway in the acquisition and retrieval of extinction using a similar auditory fear conditioning paradigm. They optogenetically stimulated ventromedial PFC (vmPFC) terminals in the amygdala during a partial extinction procedure. Although stimulation did not facilitate the within-session extinction, stimulated animals exhibited enhanced extinction retrieval the following day. In contrast, silencing vmPFC terminals during extinction impaired extinction retrieval the next day. Stimulating or silencing this projection during the extinction retrieval test did not affect conditioned freezing. These results suggest that vmPFC → amygdala projections are crucial for encoding extinction memories.

### 4.2 Prefrontal-hippocampal circuits in extinction retrieval and relapse

Despite the well-known role of the IL to amygdala projection in extinction learning, this pathway seems to be an incomplete account for the retrieval of extinction memories−specifically, it does not address the context-specificity of extinction memories. As explained above, extinction memories are highly context-dependent (Bouton, 2004; Bouton et al., 2021; Maren et al., 2013), and therefore, retrieval of extinction memories requires context information. The mPFC has connections with many other brain regions, including the HPC, which plays a crucial role in the encoding of context information, but the HPC also appears to play a role in representing extinction learning. Fear acquisition memories and extinction memories are represented in part by the dorsal DG of the HPC, and unique ensembles of DG cells represent acquisition of extinction memories (Lacagnina et al., 2019). DG activity is necessary for the expression of an extinction memory (Denny et al., 2014; Bernier et al., 2017; Lacagnina et al., 2019) and is encoded through distinct ensembles of cells that fire concurrently during extinction retrieval.

It is well established that the HPC is important for the contextual specificity of extinction and that it is in turn responsible for low levels of freezing when retrieval occurs in the extinction context, but at the same time, promotes renewal of extinguished fear (Corcoran and Maren, 2001; Hobin et al., 2006; Zelikowsky et al., 2013; Jin and Maren, 2015b; for a review see Bouton et al., 2006a). As mentioned above, vHPC preferentially sends strong projections to PV-expressing inhibitory interneurons in the IL, thus promoting fear renewal through feedforward inhibition of IL principal cells (Marek et al., 2018). On the other hand, there is evidence that ventral hippocampal projections release brain-derived neurotrophic factor (BDNF) in the IL, which appears to be sufficient for extinction learning (Peters et al., 2010; Rosas-Vidal et al., 2014), suggesting that hippocampal modulation of the IL is dynamic, and its behavioral effects can vary depending on the neuronal subtype and whether extinction has already taken place or not (Figure 1).

Considering the context-dependence of extinction memories (Maren et al., 2013; Bouton et al., 2021), extinction retrieval may involve mPFC-HPC communication. HPC-dependent episodic memories might require mPFC-dependent rules, executive function, and outcome expectancies for successful memory retrieval (Dolleman-van der Weel et al., 2019). Indeed, it is proposed that mPFC exerts a top-down control on HPC during retrieval of episodic memories by suppressing competing context-inappropriate memories and retrieving the context-appropriate one (Eichenbaum, 2017). This would imply that the retrieval of extinction requires both the suppression of the CS memory from the conditioning context (Context A) and the retrieval of the CS memory from the extinction context (Context B). This theory is also consistent with the fact that extinction creates a new inhibitory memory on top of conditioning memory that competes with the conditioning memory to be retrieved (Quirk and Mueller, 2008; Bouton et al., 2021).

### 4.3 Thalamic nucleus reuniens mediates extinction encoding and retrieval

Although the mPFC interacts with the HPC during extinction retrieval, there are no direct projections from the mPFC to HPC (but see Malik et al., 2022). However, there is considerable data revealing bidirectional projections of the midline thalamic RE with both the mPFC and HPC (Vertes, 2006; Padilla-Coreano et al., 2012; Anderson et al., 2016; Place et al., 2016; Eichenbaum, 2017; Ramanathan et al., 2018a; Dolleman-van der Weel et al., 2019; Vertes et al., 2022). In addition to the mPFC and HPC, the RE receives input from a diverse set of regions including the hypothalamus, amygdala, basal forebrain, and the brainstem (McKenna and Vertes, 2004). However, its projections are limited to limbic cortical areas (Vertes, 2006; Vertes et al., 2006). Importantly, the bidirectional connections RE has with both mPFC and HPC (Vertes, 2006; Vertes et al., 2022) positions it to coordinate information flow between the two. In addition, ∼5–10% of RE cells project to both mPFC and HPC via axonal collaterals (Hoover and Vertes, 2012). Vertes et al. (2007) combined anterograde and retrograde tracing methods to decipher mPFC-RE-HPC projections and showed that mPFC fibers form excitatory synapses on proximal dendrites of RE cells that project to the CA1 of HPC. It is known that glutamate is the primary excitatory neurotransmitter in the RE (Bokor et al., 2002), and that there are no GABAergic inhibitory interneurons (Ottersen and Storm-Mathisen, 1984). However, GABA receptors are present in the RE (Hallock et al., 2016; Walsh et al., 2017; Viena et al., 2018) and there are robust GABAergic projections from the RE to the zona incerta, for example (Yang et al., 2022). It should also be noted that RE predominantly projects to vHPC compared to dHPC (Su and Bentivoglio, 1990; Dolleman-Van Der Weel and Witter, 1996; Hoover and Vertes, 2012). Hauer et al. (2019) demonstrated that paired-pulse stimulation of mPFC or optogenetic activation of RE causes evoked potentials in the CA1 region of HPC. Evoked potentials following mPFC stimulation had longer latency compared to those following RE activation and stimulating mPFC caused orthodromic excitation in RE units. Moreover, stimulating mPFC and chemogenetically silencing RE at the same time abolished evoked potentials in HPC, supporting that RE is an intermediary structure for mPFC → HPC information flow (Hauer et al., 2019).

Evidence describing the dense projections between the mPFC, RE, and HPC paved the way for investigations of the role of the RE in the acquisition and retrieval of extinction. In a series of experiments, Ramanathan et al. (2018a) used the GABA_*A*_ receptor agonist muscimol to reversibly inhibit the RE prior to various stages of auditory fear conditioning, extinction, and extinction retrieval. Local muscimol infusions into the RE prior to extinction training impaired acquisition of extinction, and the same rats tested off-drug showed a deficit in extinction retrieval. Similarly, rats that received muscimol in RE after extinction training and immediately prior to extinction retrieval showed increased CS-induced freezing. However, when rats were tested for renewal in the conditioning context, freezing was unaffected. In addition, extinction retrieval increased Fos expression in RE compared to home-cage controls, and single-unit recordings from RE neurons during retrieval and renewal revealed increased CS-evoked firing during extinction retrieval but not renewal. These results suggest that RE has a role in inhibiting conditioned fear during extinction and extinction retrieval (Figure 1). However, the RE appears to have negligible effect on the consolidation or reconsolidation of extinction memories (Vasudevan et al., 2022). Vasudevan et al. (2022) showed that muscimol infusions in RE immediately after extinction training do not impair consolidation of extinction. Likewise, RE muscimol infusions immediately after the reactivation of the extinction memory did not impair reconsolidation of extinction. Overall, these findings suggest a selective role of the RE in encoding and retrieval processes that are involved in extinction memories.

The neural projections mediating this effect originate in the mPFC. Using an intersectional method to manipulate mPFC neurons projecting to the RE, Ramanathan et al. (2018a) found that chemogenetic silencing of RE-projecting mPFC neurons also produced an extinction retrieval deficit. Using a complementary approach, it was found that chemogenetic silencing of mPFC terminals in the RE led to a similar extinction retrieval impairment. This work suggests that mPFC to RE projections are, in part, responsible for modulating the expression of extinction memories and that the mPFC mediates top-down control for fear inhibition via the RE (Ramanathan et al., 2018a).

More recently, Ratigan et al. (2023) demonstrated that silencing projections from RE to dorsal CA1 also impaired the extinction of contextual fear conditioning in head-fixed mice. Two-photon imaging of RE terminals in CA1 revealed an increase in calcium activity during bouts of freezing and a decrease in activity during running. Collectively, this work suggests that the RE may serve as a hub interconnecting the mPFC and HPC to regulate the suppression of context-inappropriate memories (i.e., retrieval suppression) (Figure 1). This is consistent with work in humans suggesting that tasks that require participants to actively suppress memory (e.g., think-no think) result in increased activity in the PFC and reduced activity in the HPC (Anderson and Floresco, 2022). Indeed, we have recently found that RE coordinates oscillatory synchrony in the mPFC and HPC during extinction retrieval, and that recruiting this activity can prevent relapse (Totty et al., 2023). Recently, Malik et al. (2022) also revealed a monosynaptic long-range inhibitory projection from mPFC to dHPC though the role of this projection in extinction retrieval has not been explored.

Notably, recent work suggests that projections from RE to the BLA may underlie the extinction of remote fear memories. Silva et al. (2021) found that the extinction of 30-day old fear memories, but not 1-day old memories, recruited RE → BLA projections. Calcium imaging experiments revealed that RE activity and the activity of RE → BLA projections were both highly correlated with freezing behavior and increases in RE activity anticipate the cessation of freezing behavior. Closed-loop inhibition of the RE at the beginning of a freezing bout prolonged freezing behavior suggesting that RE normally contributes to the cessation of freezing behavior and may signal safety to the BLA (Silva et al., 2021). In contrast, two-photon recordings from RE terminals in dorsal CA1 showed the opposite pattern of activity during the retrieval of a contextual fear memory: RE terminals showed increases in activity during freezing and a decrease in activity during running (Ratigan et al., 2023). These authors concluded that the RE-CA1 projection suppresses fear by disrupting hippocampal contextual fear memory. Moscarello (2020) proposed a similar role for vmPFC-RE projection in freezing suppression following signaled active avoidance training, facilitating adaptive proactive coping behavior.

### 4.4 Thalamic modulation of mPFC-HPC communication during extinction retrieval

Several models have been put forward regarding the role of RE in modulating mPFC-HPC communication. Dolleman-van der Weel et al. (2019) propose that the RE may facilitate mPFC-HPC coordination via its collateralized axons targeting both mPFC and HPC. In addition, RE may integrate mPFC and other inputs to later project to HPC. This way, mPFC control over HPC processes might be exerted to regulate the retrieval of specific HPC-dependent memories. In addition, RE may gate HPC projections to mPFC and control the flow of contextual information to the mPFC (Dolleman-van der Weel et al., 2019). According to the model put forward by Eichenbaum (2017), contextual cues are delivered to mPFC directly from vHPC with RE facilitating synchrony between the two. When a specific memory is to be retrieved using these contextual cues, RE synchronizes information flow from mPFC to HPC. The mPFC, in turn, exerts top-down control over HPC and suppresses context-inappropriate HPC representations.

One way the RE may facilitate information exchange between the mPFC and HPC is via oscillatory synchrony (Ferraris et al., 2018; Hauer et al., 2019, 2021; Angulo-Garcia et al., 2020; Cassel et al., 2021). Oscillations enable communication between brain regions by synchronizing their activity (Fries, 2015; Totty and Maren, 2022). For example, theta oscillations (4–12 Hz) in the amygdala are known to couple with mPFC and HPC during the retrieval of conditioned fear memories (Seidenbecher et al., 2003; Lesting et al., 2011; Likhtik et al., 2014; Stujenske et al., 2014; Davis et al., 2017; Ozawa et al., 2020). However, not much is known about the oscillatory correlates of extinction and extinction retrieval (Lesting et al., 2013; Trenado et al., 2018; Totty and Maren, 2022). There is some evidence that disrupting hippocampal theta oscillations impairs memory retrieval (Shirvalkar et al., 2010; Etter et al., 2023). Many studies also showed that mPFC and HPC are coupled to each other at theta frequencies during different memory tasks (Jones and Wilson, 2005; Siapas et al., 2005; Hyman et al., 2010; Colgin, 2011; Lesting et al., 2011, 2013; O’Neill et al., 2013; Totty and Maren, 2022; Stout et al., 2023; Totty et al., 2023). Importantly, Lesting et al. (2011) showed that IL synchronizes with dHPC at theta frequencies during extinction retrieval in mice. They further showed that IL theta leads HPC theta during extinction retrieval but not after extinction training. Moreover, during extinction retrieval, there is no significant lead/lag relationship in oscillations between HPC-lateral amygdala and IL-lateral amygdala pairs (Lesting et al., 2013), supporting the idea that mPFC-HPC theta coupling, with mPFC leading HPC, might underlie extinction retrieval. Totty et al. (2023) similarly demonstrated mPFC-dHPC theta coupling (6–8 Hz) in the rat during the retrieval of extinction memories. This coupling was observed for both PL-HPC and IL-HPC pairs.

Based on these results, theta oscillations might facilitate inter-regional communication during the retrieval of extinction memories. Consistent with this, compelling data from Hallock et al. (2016) showed a causal role of RE in driving mPFC-HPC oscillatory synchrony during a working memory task. They observed mPFC-dHPC oscillatory synchrony during a spatial working memory task in rats, which was abolished with muscimol infusions in the RE. Kafetzopoulos et al. (2018) recorded local field potentials (LFPs) from PL and CA1 of the vHPC in RE-lesioned or sham-operated anesthetized rats. Although RE lesion did not reduce activity in the mPFC and the vHPC, it decreased mPFC-vHPC coupling in delta and theta bands compared to sham-operated rats. Totty et al. (2023) more directly showed theta-range oscillations in the RE by recording LFPs from the RE during extinction training. Moreover, pharmacological inactivation of the RE impaired both mPFC-dHPC theta coupling and extinction retrieval.

However, some studies contradict the findings described above. Roy et al. (2017) recorded LFPs from PFC, HPC, and RE in anesthetized rats. Pharmacologically inactivating RE had minimal effects on PFC-HPC theta coherence. This led authors to conclude that RE has no role in transmitting theta oscillations between PFC and HPC (Roy et al., 2017). Jayachandran et al. (2023) showed mPFC-HPC coherence in the beta band (15–30 Hz) during a non-spatial sequence memory task, while theta coherence was observed during non-memory-related running. Activating RE increased mPFC-HPC beta coherence while decreasing theta coherence (Jayachandran et al., 2023). It should be noted that these studies differ in both the behavioral tasks and recording methods (e.g., awake vs. anesthetized animals) used in the work. More work is needed to clarify the role of the RE in mPFC-HPC oscillatory coupling and how this might facilitate extinction retrieval.

In short, RE appears to contribute to mPFC top-down control of HPC-dependent memory retrieval, and this may be central to the suppression of fear during extinction retrieval. There is considerable evidence that the thalamic RE is an important hub between the mPFC and HPC and is required for the acquisition and retrieval of extinction memories (Figure 1). Whether the RE facilitates the mPFC-HPC communication through oscillatory synchrony during extinction retrieval should be examined in depth.

## 5 Stress-induced impairments in extinction acquisition

Successfully encoding and retrieving extinction memories is critical not only for the adaptive regulation of fear, but also for therapeutic interventions in patients with stress- and trauma-related disorders, such as PTSD. Work over the past two decades has revealed that extinction learning and memory are highly sensitive to stress (Maren and Holmes, 2016). When experienced before extinction, stress undermines extinction learning and causes poor long-term extinction memory. When experienced after extinction learning, stress drives the relapse of fear and impairs extinction retrieval (Maren and Holmes, 2016).

A wide variety of acute and chronic stressors including electric shock, restraint, social defeat, immobilization, and predator exposure impair extinction learning and retrieval. Additionally, there are stress models, such as the immediate-extinction deficit (IED) (Maren and Chang, 2006), stress-enhanced fear learning (SEFL) (Rau et al., 2005), and single prolonged stress (SPS) (Liberzon et al., 1997) that recapitulate physiological and behavioral changes seen in patients with PTSD. In the remaining sections, we will discuss the effects of these stressors and stress models on the mPFC, HPC, and their interaction during the regulation of fear.

### 5.1 Role of the mPFC in stress-induced extinction impairments

Substantial data indicate that the mPFC is central to the pathophysiology of PTSD (for a review see Liberzon and Sripada, 2008). This has been modeled in rodents using procedures such as SPS, in which rats experience a 2-h restraint period followed by a 20-min forced swim, exposure to ether until loss of consciousness, and then a 7-day quiescent period (Liberzon et al., 1997). After this 7-day quiescent period, SPS rats show deficits in extinction retrieval that are analogous to deficits seen in patients with PTSD (Knox et al., 2012). Considerable work has found that SPS causes lasting hypofunction in IL (Knox et al., 2010; Lim et al., 2017; Piggott et al., 2019; Nawreen et al., 2021), and this is associated with extinction retrieval deficits (Canto-de-Souza et al., 2021; Omura et al., 2021).

The IED demonstrates stress-induced deficits in extinction learning which occur when extinction learning takes place immediately after conditioning. This deficit does not seem to emerge during the extinction sessions as within-session decrement in freezing is similar between groups undergoing extinction immediately (15-min) or 24 h after conditioning (Maren and Chang, 2006). However, when rodents are tested for extinction retrieval, those submitted to extinction immediately after conditioning exhibit robust deficits in extinction retrieval. Although the mechanisms are not completely understood, evidence points to the importance of footshock-induced stress associated with Pavlovian fear conditioning (Maren, 2014, 2022). Indeed, we have demonstrated that fear conditioning (5 shock-tone pairings) causes a substantial, but short-lived, increase in spontaneous firing rates of neurons in the PL and IL. Immediately after fear conditioning, however, the increase in IL neuronal activity is followed by a sustained suppression of spontaneous firing rate for roughly 30 min after the last CS-US pairing−which includes the time window in which immediate extinction training begins (Fitzgerald et al., 2015; Giustino et al., 2016a,^2019^).

The locus coeruleus-norepinephrine (LC-NE) system is implicated in stress-induced hyperarousal and sensitization of this system is linked to PTSD (Southwick et al., 1997, 1999; Geracioti et al., 2001). Propranolol, a non-selective β-adrenergic receptor antagonist, has been used to prevent or treat symptoms of PTSD, such as hyperarousal (Southwick et al., 1999; Giustino et al., 2016b) and may promote extinction. Consistent with this, systemic propranolol administration attenuates shock-induced changes in mPFC spike firing and prevents the IED (Fitzgerald et al., 2015). Interestingly, propranolol attenuated the IED when infused into to the BLA, but not the IL, suggesting that heightened NE signaling in the BLA is a critical substrate of the IED (Giustino et al., 2017). Consistent with this, Giustino et al. (2020) discovered that fear conditioning (and the stress it engenders) produced a prolonged increase in BLA spontaneous firing that was blocked by systemic propranolol. Basolateral amygdala hyperexcitability and the IED were enabled by chemogenetic activation of the LC and these effects were blocked by propranolol (Giustino et al., 2020). Collectively, these results support the hypothesis that noradrenergic LC-BLA signaling in the BLA underlies the IED. Stress-induced activation of LC-NE system and resultant increases in BLA activity may strengthen BLA-mediated suppression of the IL, causing impairments in extinction learning (Maren, 2022) (Figure 1).

One possible mechanism in which the BLA may drive stress-induced IL neuronal activity decrease is through the activation of inhibitory interneurons. Indeed, PV^+^ interneurons provide fast-spiking inhibitory signals to mPFC neurons (Tremblay et al., 2016), constituting a possible target of acute stress effect. Page et al. (2019) showed that chronic stress increases the activity of prefrontal PV^+^ cells and that the chemogenetic activation of these cells is involved in anxiety-like behavior. We have recently shown that chemogenetic excitation of IL PV^+^ neurons during delayed extinction (24 h after fear conditioning) induces extinction learning impairments (Binette et al., 2023). Chemogenetic excitation of IL PV^+^ neurons may mimic the conditions associated with shock-induced suppression of IL activity, thereby causing extinction learning impairments. In support of this hypothesis, chemogenetic inhibition of IL PV^+^ neurons attenuated the IED in male rats (though female rats did not show an attenuated IED) (Binette et al., 2023). Moreover, chemogenetic inhibition of BLA neurons projecting to the IL prevented the IED in male rats, suggesting that the BLA may drive feed forward inhibition of the IL through its connections to IL PV + interneurons (Binette et al., 2023). Taken together, these data point to a broader circuit model in which stress induces activation of LC-NE projections to the BLA, enhancing BLA-mediated feedforward inhibition of the IL through activation of the IL PV + interneurons, ultimately leading to deficits in extinction learning (Maren, 2022) (Figure 1).

### 5.2 Role of the HPC in stress-induced extinction impairments

The discovery of steroid hormone receptors in HPC neurons (McEwen et al., 1968), especially mineralocorticoid and glucocorticoid receptors (MR and GR, respectively) in the HPC (de Kloet et al., 1996, 1998) suggest that they play a critical role in stress effects on memory (for a review see Kim and Diamond, 2002). The HPC receives inputs from sensory and association areas, and its outputs innervate cortical and subcortical areas involved in cognitive, affective, and behavioral functions (Bienkowski et al., 2018). It is therefore a critical hub for processing interoceptive and exteroceptive contexts and regulating affective behavior (Maren et al., 2013). HPC neurons are highly plastic and vulnerable to stress. For example, stressors have been demonstrated to suppress adult neurogenesis, inhibit the survival of newborn cells, and induce atrophy within the HPC (McEwen, 1999; Vyas et al., 2002; Krugers et al., 2010). These stress-induced adaptations may be due to glucocorticoid-glutamatergic interactions (Karst and Joëls, 2003, 2005; Olijslagers et al., 2008). Indeed, SPS induces an upregulation of GRs and alterations in NMDAR mRNA in the HPC, both effects that are thought to mediate the stress-induced extinction retrieval deficit. Two hours of acute restraint stress promotes fear extinction deficits in a contextual fear conditioning paradigm and AMPA-GluA1 phosphorylation in HPC neurons after 10 days of incubation (Novaes et al., 2021). These data suggest an important stress effect on HPC neurons with implications for the fear extinction process, however, more work needs to be done to determine the exact role of the HPC in stress-induced extinction deficits (Figure 1).

As discussed previously, the communication between mPFC and HPC is fundamental for fear learning and extinction. Knowing that both structures are sensitive to stress-induced plastic effects with important implications in the fear extinction process, it is important to understand whether stress-induced fear extinction impairments are correlated with alterations in mPFC-HPC interactions. Chronic stress is known to block long-term potentiation (LTP) on the vHPC-mPFC pathway (Cerqueira et al., 2007), whereas blocking extinction-induced LTP by low-frequency stimulation of vHPC neurons after extinction training disrupts the recall of extinction memory (Hugues and Garcia, 2007). Indeed, Garcia et al. (2008) showed that chronic stress disrupts the extinction memory recall by blocking the extinction-induced HPC-mPFC pathway LTP. Although the mechanisms through which stress induces changes in mPFC-HPC communication are not completely understood, evidence points to a stress-induced decrease in BDNF release in mPFC and HPC (Solomon et al., 2019). These results demonstrate that blockade of extinction-induced LTP in HPC-mPFC pathway and the decrease of BDNF release in both structures are pieces of the mechanism through which chronic stress induces fear extinction recall impairments. Understanding how stress induces changes in neurobiological substrates of extinction acquisition, such as HPC and mPFC, is fundamental to develop future therapies to stress-related psychiatric disorders.

## 6 Summary

Understanding the underlying brain circuitry in fear and extinction memory is key for increasing treatment efficiency for people with fear, anxiety, and trauma and stressor-related disorders. Considerable work reveals that the conditioning and extinction of fear requires a neural circuit involving the HPC, mPFC, and amygdala. The reviewed literature suggests that the inhibition of conditioned fear after extinction is mediated by the suppression of HPC-dependent fear memories. This is mediated by medial prefrontal cortical regulation of hippocampal fear memory retrieval, a process that is coordinated by the midline thalamic RE (Figure 1).

Successful extinction learning and retrieval is fundamental to cognitive-behavioral interventions for disorders including PTSD. A major challenge to these therapies is relapse, in which extinguished fear returns under many conditions. Psychological stress is a major contributor to relapse insofar as stress undermines both extinction learning and retrieval. Stress-induced extinction impairments appear to involve several neuromodulatory pathways, and recent work suggests that stress-induced noradrenergic hyperarousal plays a central role. Stress-induced activation of the locus coeruleus leads to amygdala hyperexcitability which, in turn, dysregulates medial prefrontal cortical circuits necessary for extinction learning (Figure 1).

Collectively, decades of research have now revealed discrete neural circuits that are central to fear conditioning and extinction. This work is opening new avenues of research to inform how failures of extinction learning and memory contribute to pathological disorders, such as PTSD.

## Author contributions

SP: Writing – original draft, Writing – review and editing. TT: Writing – original draft, Writing – review and editing. HB: Writing – original draft, Writing – review and editing. VJ: Writing – original draft, Writing – review and editing. SS: Writing – original draft, Writing – review and editing. AA: Writing – original draft, Writing – review and editing. JH: Writing – original draft, Writing – review and editing. SM: Funding acquisition, Writing – original draft, Writing – review and editing.
